# Phase II Trial of Cabozantinib Plus Erlotinib in Patients With Advanced Epidermal Growth Factor Receptor (*EGFR*)-Mutant Non-small Cell Lung Cancer With Progressive Disease on Epidermal Growth Factor Receptor Tyrosine Kinase Inhibitor Therapy: A California Cancer Consortium Phase II Trial (NCI 9303)

**DOI:** 10.3389/fonc.2019.00132

**Published:** 2019-03-11

**Authors:** Karen L. Reckamp, Paul H. Frankel, Nora Ruel, Philip C. Mack, Barbara J. Gitlitz, Tianhong Li, Marianna Koczywas, Shirish M. Gadgeel, Mihaela C. Cristea, Chandra P. Belani, Edward M. Newman, David R. Gandara, Primo N. Lara

**Affiliations:** ^1^City of Hope Comprehensive Cancer Center, Duarte, CA, United States; ^2^University of California Davis Comprehensive Cancer Center, Sacramento, CA, United States; ^3^USC Norris Comprehensive Cancer Center, Los Angeles, CA, United States; ^4^Karmanos Cancer Institute, Wayne State University, Detroit, MI, United States; ^5^Penn State Hershey Cancer Institute, Hershey, PA, United States

**Keywords:** non-small cell lung cancer, EGFR, MET, RET, VEGF, TKI resistance

## Abstract

**Introduction:** Mesenchymal epidermal transition and vascular endothelial growth factor pathways are important in mediating non-small cell lung cancer (NSCLC) tumorigenesis and epidermal growth factor receptor (EGFR) tyrosine kinase inhibitor (TKI) resistance. We hypothesized that treatment with cabozantinib plus erlotinib in *EGFR* mutation-positive NSCLC following progression on EGFR TKI therapy may allow tumors to overcome this resistance or restore sensitivity to therapy regardless of T790M status.

**Methods:** Patients with advanced NSCLC, known *EGFR* mutation and progressive disease on an EGFR TKI immediately prior to enrollment without intervening therapy were enrolled. Patients received erlotinib 150 mg and cabozantinib 40 mg daily. The primary endpoint was evaluation of efficacy by objective response rate. Secondary endpoints included assessment of progression free survival (PFS), overall survival, change in tumor growth rate, safety and toxicity, and the evaluation of specific *EGFR* mutations and *MET* amplification in pre-treatment tissue and plasma.

**Results:** Thirty-seven patients were enrolled at 4 centers. Four patients had partial response (10.8%) and 21 had stable disease (59.5%). A greater than 30% increase in tumor doubling time was observed in 79% of assessable patients (27/34). Median PFS was 3.6 months for all patients. Diarrhea (32%) was the most common grade 3 adverse event; 3 patients had asymptomatic grade 4 elevation of amylase and lipase.

**Conclusions:** Combination erlotinib and cabozantinib demonstrates activity in a highly pretreated population of patients with *EGFR* mutation and progression on EGFR TKI. Further elucidation of beneficial patient subsets is warranted.

**Clinical Trial Registration:** www.ClinicalTrials.gov, identifier: NCT01866410

## Introduction

The search for improved therapies in NSCLC has led to the investigation of agents that target novel pathways involved in tumor proliferation, invasion, and survival. EGFR signaling activates a pathway that promotes tumor proliferation, migration, stromal invasion, neovascularization, and resistance to apoptosis (1). A subgroup of patients with NSCLC has specific mutations in the *EGFR* gene that correlate with clinical responsiveness to EGFR TKI therapy (2–4). *EGFR* mutations lead to increased growth factor signaling and confer susceptibility to the inhibitor, and improved progression-free survival (PFS) when used as first-line therapy in advanced NSCLC (5, 6).

However, not all NSCLC patients with *EGFR* mutations respond to EGFR TKI therapy, and for those who initially respond to therapy, secondary resistance eventually develops (7). A specific EGFR mutation, T790M in exon 20, which develops after first- or second-generation EGFR TKI therapy is found in approximately 60% of patients with acquired resistance (8), and T790M can be accompanied by *mesenchymal epidermal transition (MET*) amplification (9). Focal amplification of the *MET* proto-oncogene promotes acquired EGFR TKI resistance in 5–20% of cases (10–14), rendering MET a potential target. The unmet needs of this patient population prompted the evaluation of cabozantinib with erlotinib. The primary targets of cabozantinib are MET and vascular endothelial growth factor receptor 2 (VEGFR2); additional targets include RET, AXL, KIT, and TIE-2 (15). The discovery of the role of angiogenesis in tumorigenesis and metastasis has paved the way for the investigation of novel antiangiogenic therapies in *EGFR* mutant NSCLC. The combination of EGFR TKI therapy and vascular endothelial growth factor (VEGF) inhibition was evaluated in a phase II trial with erlotinib and bevacizumab vs. erlotinib alone, and found a significant improvement in PFS for the combination (16), implying a benefit to simultaneous blockage of both the EGFR and VEGF pathways. The randomized phase III trial, confirmed an improved PFS with the combination (17).

A phase I/II trial evaluated the combination of erlotinib and cabozantinib in EGFR mutant NSCLC and determined the treatment to be tolerable with some clinical activity (18). A phase II study in EGFR wild type NSCLC showed that single agent cabozantinib and combination cabozantinib and erlotinib had improved PFS over erlotinib alone (19). This study builds upon the previous experience with the combination to evaluate response in patients with *EGFR* mutant NSCLC who progressed on prior EGFR TKI.

## Materials and Methods

### Eligibility Criteria

Eligible patients were required to have NSCLC harboring an *EGFR* mutation with tissue available for retrieval. Patients must have received prior EGFR TKI therapy for metastatic disease and had documented evidence of radiologic disease progression while on EGFR TKI as treatment immediately prior to enrollment, retreatment with EGFR TKI following intervening therapies was allowed. Patients must have had an Eastern Cooperative Oncology Group performance status (ECOG PS) ≤ 1; have measurable disease according to Response Evaluation Criteria in Solid Tumors (RECIST) version 1.1; and have adequate hematologic, renal, and liver function. Key exclusion criteria included prior history of MET or HGF inhibitor therapy for the treatment of cancer; previous history of gastrointestinal ulceration, bleeding in the previous 6 months; hemoptysis or pulmonary hemorrhage within 3 months; radiographic evidence of cavitating pulmonary lesion(s); prior surgery, major within 8 weeks and minor within 4 weeks (pleural catheter placement was allowed within 7 days); active central nervous system (CNS) metastasis (treated CNS metastasis allowed); or any major medical condition that would interfere with participation. The study was approved by independent ethics review boards and in accord with an assurance filed with and approved by the Department of Health and Human Services by each site. The study was conducted according to the Declaration of Helsinki. The study was approved by the Central Institutional Review Board (CIRB) for the National Cancer Institute in Rockville, MD in accord with an assurance filed with and approved by the Department of Health and Human Services at each of the participating investigational centers. All patients provided written informed consent prior to study participation.

### Study Design and Treatment

This was a phase II trial to evaluate cabozantinib and erlotinib in patients with advanced NSCLC harboring an *EGFR* mutation who progressed following EGFR TKI therapy. Patients received cabozantinib at 40 mg orally once daily plus erlotinib at 150 mg orally once daily with a cycle length of 28 days, and radiographic evaluation was performed every 2 cycles.

Dose reduction could be performed with individual drugs based on relatedness to study drug and investigator assessment. Patients were instructed to use loperamide for the prevention of diarrhea. Dose reduction for non-hematologic AEs due to cabozantinib occurred when the AE was grade 2 or higher and did not resolve to baseline in 7–10 days, or worsened. Dose reduction due to diarrhea due to erlotinib was considered if diarrhea was grade ≥3 (despite optimal loperamide use). Dose modifications for cabozantinib were 20 mg daily at dose level−1 and 20 mg every other day at dose level−2, and erlotinib 100 mg daily at dose level−1 and 50 mg daily at dose level−2. Dose re-escalation was not permitted.

### Assessments

Radiographic assessments for tumor measurement were obtained every 8 weeks (2 cycles) to evaluate PFS, overall survival (OS), objective response rate (ORR), and disease control rate (DCR) at 8 weeks. Investigator assessed disease status was performed per RECIST v1.1. Adverse events (AEs) were assessed per the Common Terminology Criteria for Adverse Events (CTCAE version 4.0).

### Biomarker Evaluation

#### EGFR Mutation Analysis

*EGFR* mutational analysis was per local CLIA certified laboratory as a part of standard of care for patients.

#### MET Amplification Analysis

*MET* amplification was assessed in a CLIA-certified laboratory (Response Genetics Inc, Los Angeles, CA) using Fluorescence *in situ* Hybridization (FISH).

### Plasma Mutation Analysis

Blood samples were collected in EDTA tubes, and were processed within 2 h using a double spin procedure. Plasma was isolated, aliquoted, and stored at −80°C. DNA extraction and sample analysis were performed at MolecularMD using QIAmp DSP Circulating Nucleic Kit (Qiagen) and QX100 ddPCR system. DNA was analyzed for the presence of the primary mutations (L858R or Exon19del) and the T790M point mutation.

### Statistical Analysis

The primary objective for this phase II trial was to determine the efficacy of cabozantinib and erlotinib in patients with EGFR mutant NSCLC progressing on an EGFR TKI by ORR with a two-stage design. Secondary objectives included assessment of PFS and OS from start of therapy; evaluation of tumor growth rate; safety and correlation of outcomes with tumor biomarkers.

Enrollment was done in two stages. In the first stage, 12 patients were treated, and if no patients exhibited a RECIST objective response, enrollment was to be closed, unless the tumor growth rate provided sufficient evidence to proceed. If 1 or more patients had an objective response, the study was to continue enrollment to the final sample size of 37 subjects. If 4 or more of the 37 patients responded, the trial would be regarded as indicating adequate activity in tumors with *EGFR* mutations, providing other factors, such as toxicity and time to progression also appeared favorable. The probability of indicating activity by this criterion was no more than 0.10 if the underlying response rate was 5%, and it was at least 0.90 if the underlying response rate was 20%.

This study also obtained a tumor growth estimate for patient's disease on the last erlotinib-based therapy. As patients progressed on treatment on prior EGFR TKI, those with at least two scans on the prior treatment to estimate the tumor growth rate were included. Tumor doubling time was estimated using an exponential growth model, providing an estimate of the tumor doubling times. Specifically, the pre-progression scan, and the baseline scan were used to estimate the doubling time prior to enrollment, td = log(2)^*^Δtime/Δlog(tumor size) [derivation, S(t) = S(t_o_)^*^2^∧^[(t−t_o_)/td] for a parameterization of exponential growth with a doubling time of td. Taking the logarithm on both sides: log(S(t))-log(S(t_o_)) = log(2)^*^(t − t_o_)/td or td = log(2)^*^(t − t_o_)/[log(S(t))-log(S(t_o_))] = log(2)^*^Δtime/Δlog(S)], and the baseline scan and the first evaluation scan were used to determine the doubling time upon protocol treatment by the same method for each patient. Based on the pre-planned protocol assessment, we estimated the percentage of patients that experienced a slowing of tumor kinetics (a 30% increase in the length of time for tumor doubling) based on RECIST v1.1 measurements. Patients who did not get a scan on study were considered as failing to demonstrate a 30% increase in doubling times, and patients whose pre-progression scans were missing or whose pre-progression tumor size was zero or whose tumor was decreasing prior to enrollment were excluded. While we recognize a natural change in the tumor growth rate at the time of treatment initiation could bias this analysis, the size, and frequency of the change in growth kinetics was considered, along with the clinical context of response, toxicity, and tolerability in both the interim and final analysis.

## Results

### Patients

**Figure 1:**
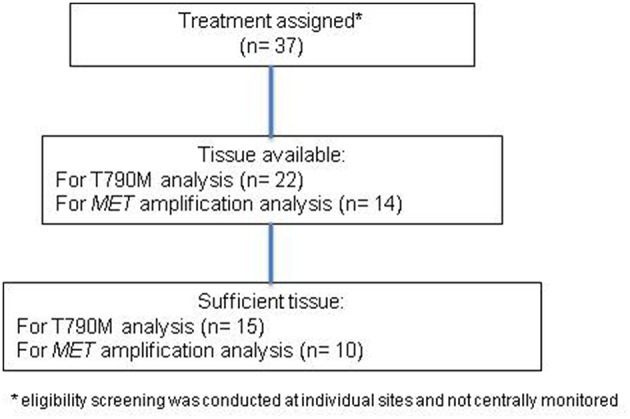
Consort diagram.

Patient characteristics with T790M mutation status are provided in Table 1. All except one patient received erlotinib as the last therapy prior to enrollment, and the remaining patient had received afatinib, while none of the patients had received osimertinib or other third-generation EGFR TKI prior to enrollment. Thirty-seven patients were enrolled (Figure 1). Median age was 64 years and the majority of patients were female. In patients with known T790M mutation status in tissue, 53% were positive for T790M. Over 40% of patients had received more than 2 prior therapies. Median follow-up was 11.0 months.

**Table 1:** Patient Characteristics.

	**All** **(*n* = 37)**	**T790M neg** **(*n* = 7)**	**T790M pos** **(*n* = 8)**	**Unknown** **(*n* = 22)**
Age, median (range)	64.6 (40.8–85.9)	64.9 (40.8–67.7)	62.3 (54.7–76.5)	64.8 (43.6–85.9)
**Gender, n (%)**				
Female	23 (62.2)	3 (42.9)	6 (75)	14 (63.6)
Male	14 (37.8)	4 (57.1)	2 (25)	8 (36.4)
**Race/Ethnicity, n (%)**				
Asian	10 (27)	3 (42.9)	3 (37.5)	4 (18.2)
Black	1 (2.7)	1 (14.3)	0	0
Caucasian (non-Hispanic)	21 (56.8)	3 (42.9)	4 (50)	14 (63.3)
Hispanic/Latino	4 (10.8)	0	1 (12.5)	3 (13.6)
Pacific Islander	1 (2.7)	0	0	1 (4.5)
**ECOG Performance Status, n (%)**				
0	19 (51.4)	3 (42.9)	4 (50)	12 (54.5)
1	18 (48.6)	4 (57.1)	4 (50)	10 (45.5)
**Histology, n (%)**				
Adenocarcinoma	26 (70.2)	3 (42.9)	7 (87.5)	16 (72.7)
Squamous cell carcinoma	1 (2.7)	1 (14.3)	0	0
NSCLC, NOS	10 (27)	3 (42.9)	1 (12.5)	6 (27.3)
**Number Of Prior Systemic Therapies**, n (%)	Median 2; range 1–8			
1	15 (40.5)	2 (28.6)	2 (25.0)	11 (50.0)
2	7 (18.9)	2 (28.6)	3 (37.5)	2 (9.1)
>2	15 (40.5)	4 (42.9)	3 (37.5)	9 (40.9)

### Efficacy

**Figure 2:**
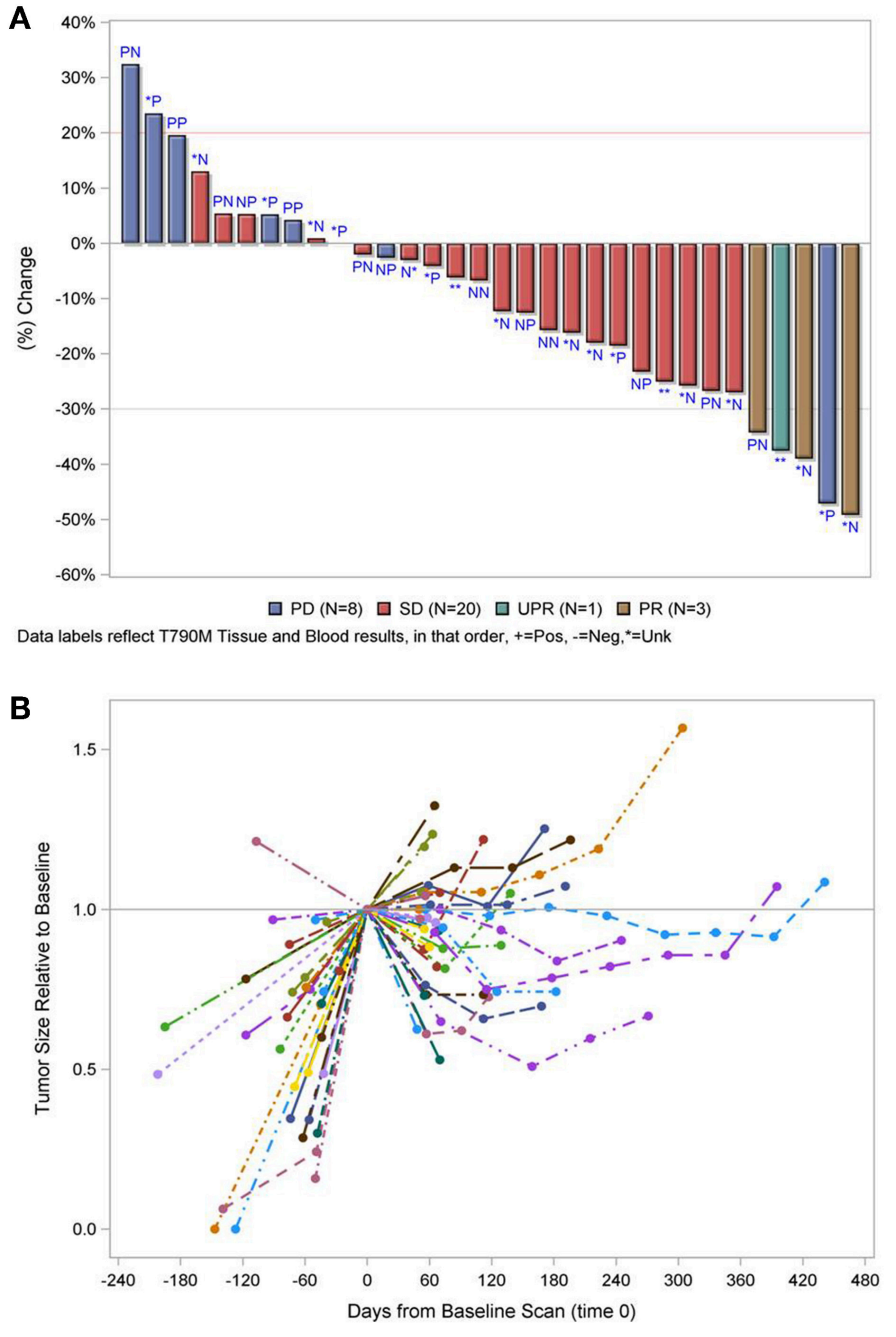
**(A)** Waterfall plot of best response by T790M mutation status in tissue and blood, in that order labeled, P, positive; N, negative; ^*^, unknown. **(B)** Relative change in tumor size. PD, progressive disease; SD, stable disease; PR, partial response. Line and color type represent individual patients. The 5 cases excluded in both figures failed therapy prior to tumor measurements. 1 with known T790M in tumor, 1 with negative T790M in blood, and three with unknown results.

**Figure 3:**
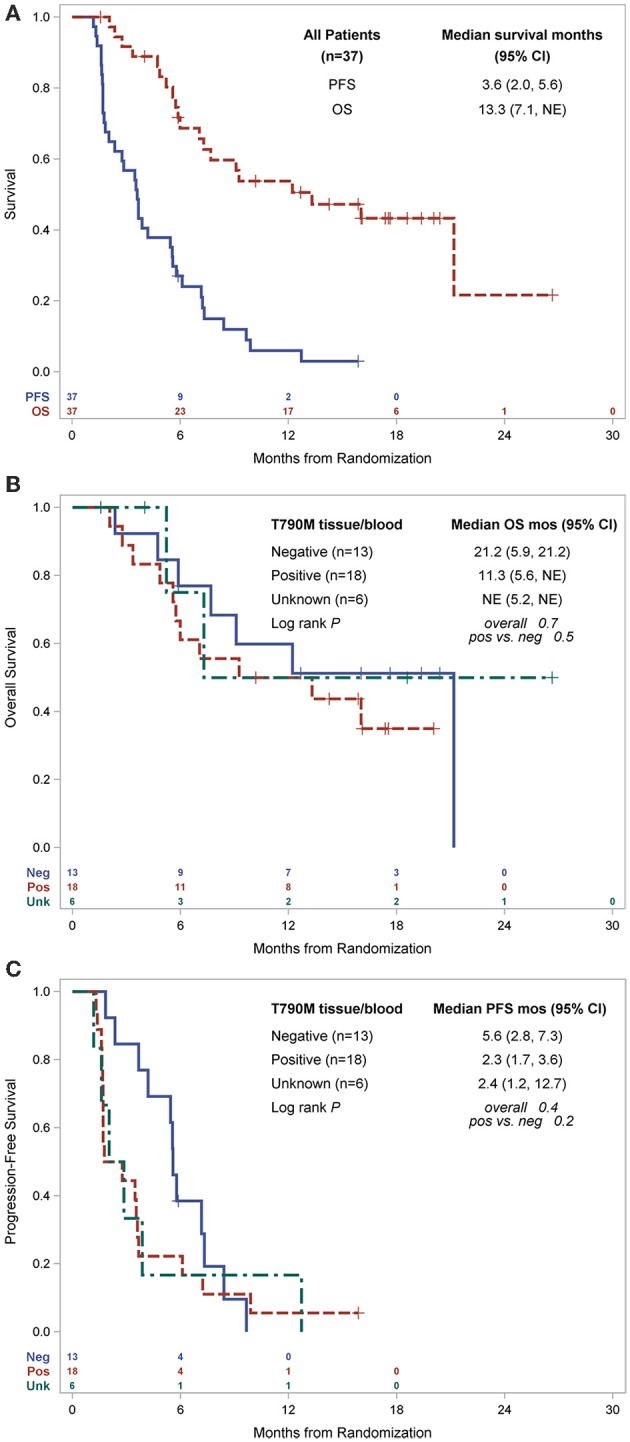
Kaplan-Meier estimates of survival, **(A)** PFS and OS for all patients and by primary *EGFR* mutation, **(B)** OS by T790M status, **(C)** PFS by T790M status. PFS, progression free survival; OS, overall survival; mo, months; CI, confidence interval; pos, positive; neg, negative; NE, not estimable.

In the overall population, the ORR was 10.8% with an 8 week DCR of 67.0% in a heavily pretreated population (Table 2). ORR was not dependent on T790M mutation status in tissue or blood (Figure 2A). A greater than 30% increase in tumor doubling time was observed in 79% (27/34) of assessable patients (95% CI: 0.62–0.91), with 3 patients excluded because of missing (*n* = 1) or size zero pre-progression scans (*n* = 1) or tumor whose size was decreasing before going on study (*n* = 1) (Figure 2B). Median PFS was 3.6 months (95% CI, 2.0–5.6 months), and median OS 13.3 was months (95% CI, 7.1-NE months) (Figure 3A). One patient remained on treatment for a year. PFS and OS were not significantly different in those with exon 19 vs. L858R *EGFR* mutations (data not shown). In addition, similar OS and PFS were noted in patients despite T790M status in tissue or blood (Figures 3B,C). *MET* amplification was evaluated, but none of the patients with available tissue had evidence of amplification at baseline.

**Table 2:** Treatment and response by mutation.

	**All** **(*n* = 37)**	**Tissue**			**Blood**		
		**T790M neg** **(*n* = 7)**	**T790M pos** **(*n* = 8)**	**Unknown** **(*n* = 22)**	**T790M neg** **(*n* = 17)**	**T790M pos** **(*n* = 12)**	**Unknown** **(*n* = 8)**
Total cycles, median (range)	4 (1–15)	4 (2–6)	3 (2–15)	3.5 (1–14)	6 (2–15)	2 (1–6)	3 (2–14)
**Off Treatment Reason, n (%)**							
Progression	33 (89.2)	6 (85.7)	7 (87.5)	20 (90.9)	14 (82.3)	12 (100.0)	7 (87.5)
Toxicity	2 (5.4)	1 (14.3)	0	1 (4.5)	1 (5.9)	0 (0.0)	1 (12.5)
Other	2 (5.4)	0	1 (12.5)	1 (4.5)	2 (11/8)	0 (0.0)	0 (0.0)
**Best Overall Response, n (%)**							
Partial response	4 (10.8%)	0	1 (12.5)	3 (13.6%)	3 (17.7)	0 (0.0%)	1 (12.5)
Progressive disease	12 (32.4)	1 (14.3)	4 (50)	7 (31.8)	2 (11.8)	7 (58.3)	3 (37.5)
ORR (95%CI)	10.8% (0.3, 21.3)	0%	12.5% (0, 42.1)	13.6% (0, 29.2)	17.6% (0, 37.8)	0	12.5% (0, 42.1)
DCR at 8 weeks (95%CI)	67.6% (51.7, 83.4)	85.7% (50.8,100)	50.0% (5.3, 94.7)	68.2% (47.0, 89.3)	88.2% (71.1, 100)	41.7% (8.9, 74.4)	62.5% (19.2, 100)
Follow-up (mo), median (range)	11.0 (1.6, 26.6)	8.6 (2.4, 16.0)	13.1 (2.8, 20.1)	11.1 (1.6, 26.6)	12.7 (2.4, 21.2)	9.2 (2.1, 17.4)	10.3 (1.6, 26.6)

### Safety

All patients who received at least one dose of the study drugs were assessed for safety (*n* = 37). The most common AEs (all grades) were diarrhea, fatigue, increase in serum amylase and rash. Diarrhea and increased amylase were the most common grade 3 AEs, and 3 patients experienced grade 4 toxicity (2 increased lipase and 1 vomiting) (Table 3). No deaths were attributable to treatment.

**Table 3:** Grade 3 or higher Toxicity.

**Adverse event**	**(*n* = 37)**	
	**Grade 3**	**Grade 4**
Alanine aminotransferase increased	1 (3%)	
Aspartate aminotransferase increased	1 (3%)	
Dehydration	2 (5%)	
Diarrhea	12 (32%)	
Fatigue	2 (5%)	
Hypertension	1 (3%)	
Hypokalemia	1 (3%)	
Hypotension	1 (3%)	
Lipase increased	2 (5%)	2 (5%)
Lymphocyte count decreased	1 (3%)	
Myalgia	1 (3%)	
Nausea/Vomiting	2 (5%)	1 (3%)
Neutrophil count decreased	1 (3%)	
Rash acneiform	2 (5%)	
Rash maculo-papular	2 (5%)	
Serum amylase increased	4 (11%)	
Thromboembolic event	1 (3%)	
White blood cell decreased	1 (3%)	

## Discussion

The combination of erlotinib and cabozantinib demonstrated clinical benefit with DCR 67% and responses seen regardless of the presence of T790M. Prolongation of tumor doubling time was observed, and treatment was tolerable in patients with *EGFR* mutation positive NSCLC who had received multiple prior therapies. Trials using targeted agents in a refractory patient population present unique challenges in design, and studies suggest that RECIST may not provide an accurate assessment of clinical benefit (20). In cases of targeted treatment, therapy should not necessarily be stopped upon documented progression if the tumor growth is still showing signs of reduction from pre-treatment values (21). In addition, in refractory populations responses are expected to be rare due to the largely cytostatic nature of the agents. These challenges highlight the need for considering novel approaches to evaluating this drug combination in this setting. In addressing these concerns, we assessed tumor growth estimate in the current study. Despite the limited ORR, the majority of patients experienced an increase in tumor doubling time, suggesting clinical benefit of combination cabozantinib and erlotinib in this heavily pretreated *EGFR* mutant population. This endpoint was prespecified to provide a more accurate assessment of clinical benefit in this population.

Currently, most patients with *EGFR* mutant NSCLC receive a first- or second-generation EGFR TKI as primary therapy for metastatic disease. In these patients, about 60% will develop T790M as a mechanism of secondary resistance (8), and another proportion will experience *MET* amplification (9). In our population, selection for *MET* amplification may have led to more dramatic responses, although we did not find *MET* amplification since only 10 patients had sufficient tissue for testing, limiting our ability to draw conclusions. In this setting, *MET* amplification has been reported in 5–20% of lung cancers with resistance to EGFR inhibitors (10, 22) Sequencing of therapy for patients with *EGFR* mutant NSCLC is in evolution, and based on Phase III results demonstrated improved PFS with osimertinib in the front-line setting for these patients (4), new mechanisms of acquired resistance will emerge. The combination of cabozantinib and erlotinib did not show differential effects based on T790M mutation status or primary *EGFR* mutation status.

Investigations of acquired resistance in patients who received first-line osimertinib have demonstrated heterogeneity in resistance mechanisms (23). Notably, in an early analysis from Massachusetts General Hospital, T790M was not found, and the most common alterations were *MET* amplification (30%) and *EGFR* C797S (22%). Ramalingam and colleagues analyzed mechanisms of resistance following front-line osimertinib or standard of care (SOC) in circulating tumor DNA (ctDNA) from the phase III FLAURA study (24). The presence of *MET* amplification was 15%, and *EGFR* C797S was seen in 7% while *MET* amplification was noted in 4% of those on SOC with 47% acquiring *EGFR* T790M. The occurrence of *MET* amplification appears to be higher in this population and will become a more important target for patients who develop resistance after osimertinib as front-line therapy. A clinical example for the use of cabozantinib and erlotinib in the setting of MET-mediated acquired resistance has also been described. A patient with an *EGFR* mutation and *MET* amplification following the development of resistance to EGFR TKI therapy experienced response to combination osimertinib and the MET inhibitor savolitinib. Upon progression, a *MET* D1228V mutation was identified and the patient developed response lasting at least 5 months to combination therapy with erlotinib and cabozantinib (25).

While cabozantinib blocks MET and VEGFR-2, it also has inhibitory activity against multiple other targets, such as RET, KIT, AXL, and TIE-2, providing additional rationale for combination therapy with erlotinib following resistance in the setting of osimertinib as front line therapy for *EGFR* mutant NSCLC. Many of the targets are rare in NSCLC and were not evaluated in this trial, growing evidence suggests the emergence of *RET* gene alterations following osimertinib and other EGFR TKI therapy as a mechanism of resistance. In an assessment of circulating tumor DNA (ctDNA) *RET* alterations in patients with cancer, 15 of 126 NSCLC patients had co-occurring *EGFR* mutation and 5 had developed resistance to prior EGFR TKI (26). None had a KIF5B fusion partner. Furthermore, response to combination therapy with osimertinib and BLU-667 was reported in a patient with acquired CCDC6-RET fusion following progression on osimertinib (27). These data suggest further potential application for cabozantinib as a multitargeted kinase inhibitor, in combination with erlotinib, following development of resistance to osimertinib or other EGFR TKIs.

Studies using cabozantinib alone and in combination with erlotinib have been performed in NSCLC. In a phase I/II trial of the combination in unselected NSCLC patient, 5 of 61 treated in the phase I portion experienced PR, while one patient in the phase II portion had PR on cabozantinib alone and none on the combination arm. As seen in the current study, diarrhea was the most common AE seen with the combination (18). Neal and colleagues performed a 3-arm, phase II randomized trial in patients with advanced non-squamous NSCLC without *EGFR* mutation (19). They found an improvement in PFS for patients who received cabozantinib alone or the combination at 4.3 and 4.7 months respectively, while PFS in the erlotinib group was 1.8 months. Furthermore, the activity of cabozantinib has been demonstrated in NSCLC harboring RET and ROS1 alterations (28, 29). Since this was a single arm combination trial, it has not clarified whether the combination is necessary for benefit or cabozantinib alone would provide similar efficacy.

Multiple mechanisms of resistance in patients with activating *EGFR* mutations are still unknown, especially with the use of osimertinib in the front-line setting. Optimal therapy following progression on EGFR TKI therapy for patients with activating *EGFR* mutations after standard TKI therapy and chemotherapy is not established (30). The activity of cabozantinib and the combination of cabozantinib and erlotinib in the changing landscape of therapy for patients with *EGFR* mutant NSCLC suggests that further evaluation in selected population may lead to benefits for patients.

## Data Availability

The datasets generated for this study are available on request to the corresponding author.

## Author Contributions

KR, PF, PM, EN, and DG: concept and design. KR, PM, BG, TL, MK, SG, MC, CB, EN, DG, and PL: collection and assembly of data. KR, PF, NR, PM, DG, and PL: data analysis and interpretation. All authors manuscript writing and final approval of manuscript.

### Conflict of Interest Statement

KR is a consultant for Genentech, Exelixis, Guardant, Loxo, Seattle Genetics, Takeda, Tesaro, and Boehringer Ingelheim, and has research support to the institution from Adaptimmune, Acea, Genentech, Zeno, Xcovery, Seattle Genetics, Boehringer Ingelheim, Takeda, BMS, Pfizer, Guardant, Loxo, and Janssen. PM is a consultant for AstraZeneca and has research support from Boehringer Ingelheim. BG is currently an employee of Genentech with stock ownership. MK received honoraria from speakers' bureau for AstraZeneca. SG is a consultant for Genentech/Roche and AstraZeneca. MC received honoraria from speakers' bureau for AstraZeneca. DG is a consultant for AstraZeneca and Genentech. PL is a consultant for Exelixis. The remaining authors declare that the research was conducted in the absence of any commercial or financial relationships that could be construed as a potential conflict of interest.
